# No change in skeletal muscle oxidative capacity biomarkers after 6 weeks of twice‐weekly single‐leg cycle interval training in young active adults

**DOI:** 10.1113/EP093493

**Published:** 2026-11-15

**Authors:** Emileigh R. Binet, Beatrice Yee, James McKendry, Changhyun Lim, Douglas L. Richards, Fiona J. Babir, Martin J. Gibala

**Affiliations:** ^1^ Department of Kinesiology McMaster University Hamilton Ontario Canada; ^2^ Food, Nutrition and Health, Faculty of Land and Food Systems University of British Columbia Vancouver British Columbia Canada; ^3^ Population Health Sciences Institute, Faculty of Medical Sciences Newcastle University Newcastle upon Tyne UK; ^4^ Department of Medicine McMaster University Hamilton Ontario Canada

**Keywords:** capillaries, interval training, mitochondria, skeletal muscle

## Abstract

Interval training (INT) of sufficient frequency, intensity and duration can increase biomarkers of skeletal muscle oxidative capacity in humans. Studies typically involve three to four sessions per week; however, the effect of reduced training frequency on this response is unclear. Most studies also do not include a non‐exercise control (CON) condition to account for the potential influence of extraneous, uncontrolled factors. We employed a single‐leg, counterweighted cycling model to investigate the effect of twice weekly INT for 6 weeks on biomarkers of skeletal muscle oxidative capacity including mitochondrial content and fibre capillarisation. Sixteen healthy active adults (6 males, 10 females; 22 ± 3 years; peak oxygen uptake: 36.1 ± 6.1 mL kg^−1^ min^−1^) performed INT with one leg and their contralateral limb served as a CON. INT involved 4 × 5‐min bouts at an intensity of 50%–60% *W*_peak_, interspersed with 2.5 min of recovery at 20% *W*_peak_. Muscle biopsy samples obtained from the vastus lateralis of both legs before and after the intervention revealed no differences in the primary outcome measure, citrate synthase maximal activity (*P* = 0.350, time × leg interaction). There were also no differences in multiple markers of mitochondrial protein content or capillarisation (all *P *> 0.05), except NDUFB8 protein content, which increased post‐training in the INT leg only (*P* = 0.0387). The lack of change in most biomarkers of oxidative capacity may be due to an insufficient training intensity or related to participant fitness or measurement variability as indicated by the typical error of ∼20%˗40% between legs as baseline.

## Abstract (highlights)

- **What is the central question of this study?**
  Does single‐leg, counterweighted cycle interval training, performed twice weekly for 6 weeks, increase biomarkers of human skeletal muscle oxidative capacity?
- **What is the main finding and its importance?**
  There was no change in the primary outcome, citrate synthase maximal activity, or most other markers of mitochondrial content and capillarisation, in the trained leg compared to pre‐training and the non‐exercise control leg. The lack of change may be owing to an insufficient training stimulus or related to baseline participant fitness or measurement variability in the biomarkers of skeletal muscle oxidative capacity.

## 1 INTRODUCTION

Regular aerobic exercise training can increase skeletal muscle oxidative capacity as reflected by biomarkers of mitochondrial content and capillarisation (Mølmen et al., 2025). Mitochondrial content is commonly assessed indirectly by measuring citrate synthase (CS) maximal activity or the protein content of the mitochondrial complexes (Larsen et al., 2012). Skeletal muscle fibre capillarisation is typically assessed using immunofluorescence labelling of endothelial cells expressing cluster of differentiation 31 (CD31^+^), a common biomarker of capillaries (Lertkiatmongkol et al., 2016). Provided the exercise stimulus is of sufficient intensity and duration, increases in biomarkers of mitochondrial content can be detected relatively rapidly, within a few training sessions, spaced ∼24–48 h apart (Egan et al., 2013; Perry et al., 2010).

Interval exercise training (INT), which typically involves short bouts of higher intensity exercise interspersed with recovery periods, can elicit increases in biomarkers of skeletal muscle oxidative capacity that are similar to or greater than traditional moderate‐intensity continuous exercise (MacInnis, Zacharewicz et al., 2017; Mølmen et al., 2025). This depends in part on whether protocols are ‘matched’ or not (i.e., based on estimates of total work or total energy expenditure between conditions) (MacInnis, Zacharewicz et al., 2017; Scribbans et al., 2014; Skelly et al., 2023). The efficacy of INT to elicit changes in muscle oxidative capacity seems mainly related to the intensity of training per se rather than the inherent alternating pattern of higher and lower intensities characteristic of intermittent exercise (MacInnis, Morris et al., 2017; Skelly et al., 2023).

Exercise training studies including most INT studies commonly involve three or more sessions per week. At this training frequency, increased biomarkers of mitochondrial content and capillarisation have been reported after 2–6 weeks in previously active individuals (MacInnis, Zacharewicz et al., 2017; Scribbans et al., 2014; Tan et al., 2018; Vincent et al., 2015). The impact of reduced training frequency (i.e., ≤2 sessions per week) on skeletal muscle oxidative capacity is unclear, but recent work showed twice‐weekly INT preformed for 6 weeks increased whole body peak oxygen uptake (${\dot{V}}_{O_{2}\mathrm{peak}}$) and performance in recreational runners (Lenk et al., 2025). It is also uncommon in studies of skeletal muscle responses to INT to include a non‐exercise, control group (CON), which otherwise helps to identify the potential influence of extraneous, uncontrolled factors and reduce potential bias.

The purpose of the present study was to investigate the effect of twice‐weekly INT, performed for 6 weeks, on skeletal muscle oxidative capacity in healthy, young, active adults. A single‐leg, counterweighted cycling model (Abbiss et al., 2011; MacInnis, McGlory et al., 2017) was used to facilitate a within‐participant design such that each participant served as their own control to help mitigate inter‐individual variability. One leg was randomly assigned to perform INT and the other leg served as a CON. The primary outcome measure was CS maximal activity. Secondary outcome measures included protein contents of the mitochondrial enzymes and markers of fibre capillarisation. We hypothesised that training would increase biomarkers of muscle oxidative capacity in the INT trained leg as compared to the same leg before training and the CON leg after training.

## 2 METHODS

### 2.1 Ethical approval and pre‐registration

Ethical approval was obtained from the Hamilton Integrated Research Ethics Board (Project no.: 16486). All participants provided written, informed consent prior to being enrolled in the study. This study was pre‐registered on the Open Science Framework site (https://osf.io/md72g/). All protocols were in accordance with the *Declaration of Helsinki* (‘Human Experimentation’, 1964).

### 2.2 Sample size and participants

An a priori sample size estimate was performed using G*Power statistical software (v. 3.1.9.7; Düsseldorf, Germany) (Faul et al., 2007). The calculation was based on a two‐factor (time × group) within‐subject, repeated measures, analysis of variance (ANOVA). It was determined that a sample size of 16 participants was required to detect a significant difference in CS maximal activity, based on previous work (MacInnis, Zacharewicz et al., 2017). The following parameters were used for the calculation: 80% power, an α‐level of 0.05, a large effect size of 0.4 and a correlation among repeated measures of 0.5.

Participants were recruited from McMaster University and the Hamilton community using hard copy and online posters. The inclusion criteria were healthy, young, active adults aged 18–35 years. This age range was selected since it is prior to the onset of age‐related declines in maximal oxygen uptake (${\dot{V}}_{O_{2}\mathrm{peak}}$) (Heath et al., 1981). Health and habitual activity status were assessed by self‐report based on the Canadian Society for Exercise Physiology (CSEP) Get Active Questionnaire (GAQ) (‘CSEP Get Active Questionnaire’, 2021). Participants were deemed active if they met CSEP's guidelines of engaging in at least 150 min of moderate‐to‐vigorous aerobic physical activity per week. Exclusion criteria included answering ‘Yes’ to any of the screening questions on the GAQ, any known cardiovascular and/or musculoskeletal conditions, and/or not being cleared by a health care practitioner to engage in physical activity.

A total of 21 participants were recruited. Sixteen participants (6 males, 10 females) completed the study. One participant (female) no longer met the inclusion criteria, one participant (female) was lost to follow‐up, and three participants (all male) declined to continue the intervention. Participant enrolment and attrition are reported in Figure 1 and were modelled in accordance with Consolidated Standards of Reporting Trials guidelines for within person randomised trials (Pandis et al., 2017). Characteristics of the participants who completed the study are summarised in Table 1.

**FIGURE 1:**
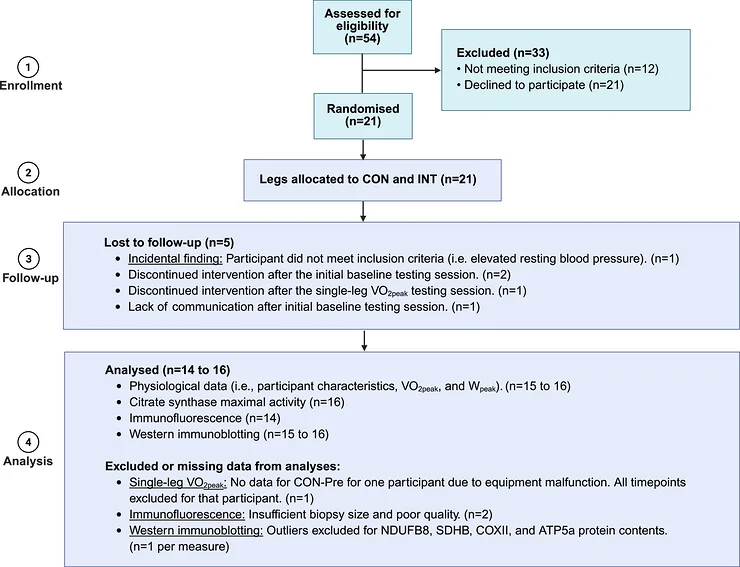
Consolidated Standards of Reporting Trials diagram. CON, non‐exercise control leg; INT, interval trained leg; ${\dot{V}}_{O_{2}\mathrm{peak}}$, peak oxygen uptake; *W*_peak_, peak power output. Created in BioRender. Gibala, M. (2026) https://BioRender.com/o5ilf5m.

**TABLE 1:** Participant characteristics.

Measure	Pre‐value
Sample size (*n*)	16; 6 males, 10 females
Age (years)	22 ± 3 (20, 23)
Height (cm)	165.1 ± 0.2 (164.9, 165.2)
Total body mass (kg)	69.3 ± 14.7 (61.5, 77.1)
Body mass index (kg m^−^^2^)	24.2 ± 3.8 (22.2, 26.3)
Double‐leg cycling ${\dot{V}}_{O_{2}\mathrm{peak}}$ test	
Absolute ${\dot{V}}_{O_{2}\mathrm{peak}}$ (L min^−^^1^)	2.5 ± 0.8 (2.1, 3.0)
Relative ${\dot{V}}_{O_{2}\mathrm{peak}}$ (mL kg^−^^1^ min^−^^1^)	36.1 ± 6.1 (32.8, 39.3)
*W*_peak_ (W)	236 ± 63 (202, 269)

*Note*: Data presented as mean ± SD (95% confidence interval lower‐ and upper‐bounds).

Abbreviations: ${\dot{V}}_{O_{2}\mathrm{peak}}$, peak oxygen uptake; *W*_peak_, peak power.

### 2.3 Experimental design

This study employed a randomised, repeated measures, within‐subject design. A single‐leg cycling exercise model was used, with each participant's contralateral limb serving as their own internal control. An overview of the study timeline is presented in Figure 2. In brief, participants completed a preliminary screening session that included anthropometric measurements and a double‐leg ${\dot{V}}_{O_{2}\mathrm{peak}}$ test. Approximately 48 h after the screening session, participants completed single‐leg ${\dot{V}}_{O_{2}\mathrm{peak}}$ tests, with the testing order randomised between legs. Muscle biopsies were performed on both legs 48 h after the single‐leg ${\dot{V}}_{O_{2}\mathrm{peak}}$ tests. Following pre‐testing, participants completed 6 weeks of INT using the leg assigned to training. Following the 6‐week intervention, participants repeated the single‐leg ${\dot{V}}_{O_{2}\mathrm{peak}}$ tests and had a second set of muscle biopsies >48 h after post‐intervention ${\dot{V}}_{O_{2}\mathrm{peak}}$ testing. Participants were instructed to maintain their habitual activity patterns throughout the study.

**FIGURE 2:**
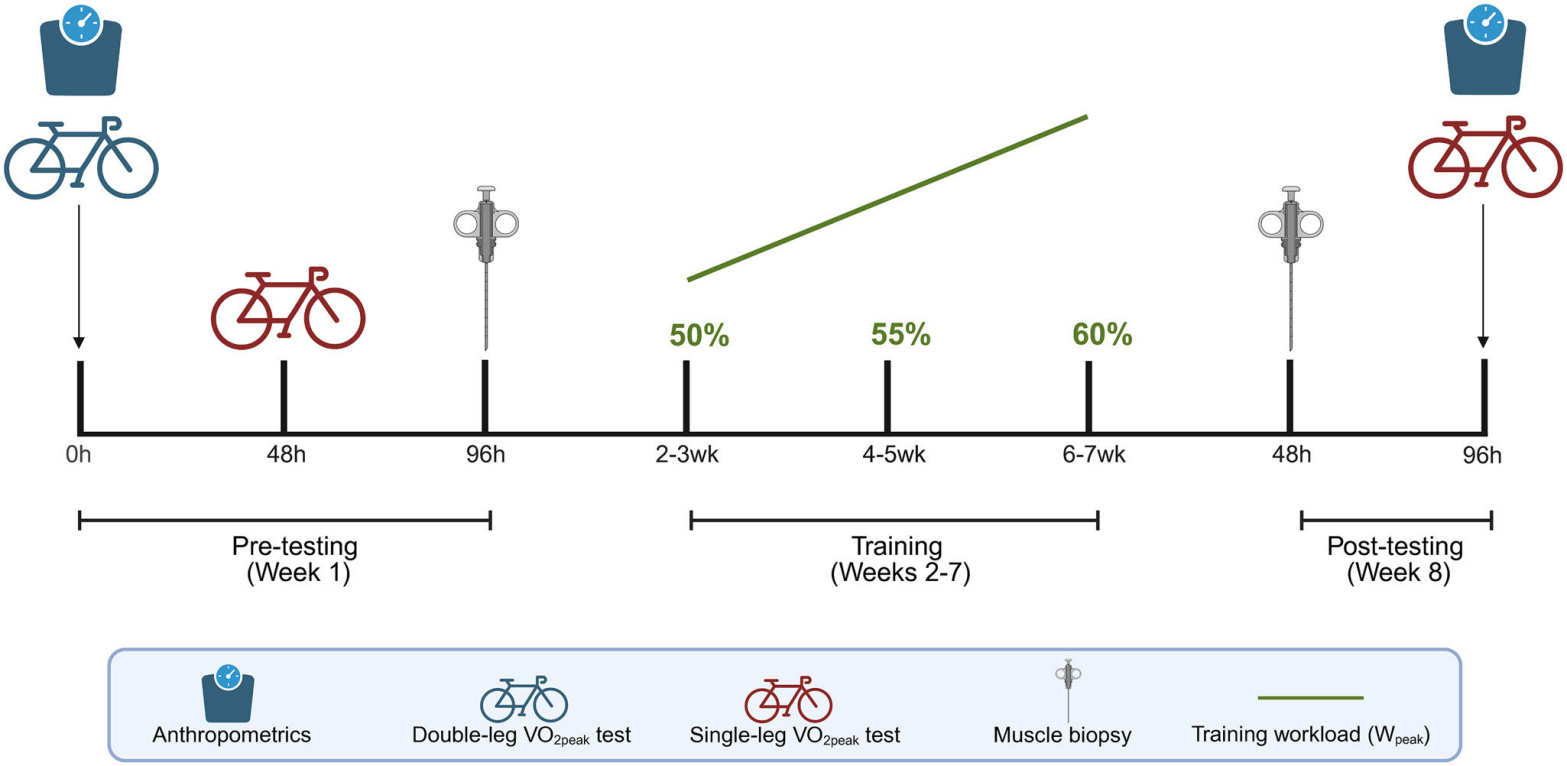
Experimental timeline. ${\dot{V}}_{O_{2}\mathrm{peak}}$, peak oxygen uptake; *W*_peak_, peak power. Created in BioRender. Gibala, M. (2026) https://BioRender.com/y4aswah.

### 2.4 Randomisation

Simple randomisation was selected to randomise the testing order of the limbs for the unilateral ${\dot{V}}_{O_{2}\mathrm{peak}}$ tests. The testing order of the limbs was determined by a coin toss performed by the investigator (i.e., tails = left leg, heads = right leg).

Block randomisation was selected to randomise participants’ legs to CON and INT. The randomisation process (Altman & Bland, 1999) is summarised as follows. A block size of four participants was chosen which evenly divides into five, blocked groups, totalling 20 participants. A total of six balanced sequences were determined based on CON or INT legs. The order of the sequences to be used for each block was determined using an online random number generator (https://www.calculatorsoup.com/calculators/statistics/random‐number‐generator.php). The left leg was chosen to be allocated to the condition first; therefore, the condition listed in the sequence was deemed the condition for the left leg, while the right leg was allocated to the opposite condition (e.g., the first condition was INT, which meant that the left leg was allocated to the exercise condition while the right leg served as the CON limb).

### 2.5 Participant characteristics and exercise capacity

Participant height and body mass were measured using a stadiometer medical scale (Pelstar‐LLC, Health o meter‐professional, 500KL; Countryside, IL, USA) during the pre‐ and post‐testing procedures. Resting blood pressure was measured using an automated monitor and cuff (Omron, BP7455CAN; Kyoto, Japan) prior to all exercise sessions. Heart rate (Polar Electro Oy, M430 watch and H9 sensor; Kempele, Finland) was measured continuously for all trials. A Borg 6–20 rating of perceived exertion (RPE) scale was used to assess participant exertion for all trials (Borg, 1970).

#### 2.5.1 ${\dot{V}}_{O_{2}\mathrm{peak}}$ tests

Double‐ and single‐leg ${\dot{V}}_{O_{2}\mathrm{peak}}$ tests were performed as previously described (MacInnis, Zacharewicz et al., 2017) to determine ${\dot{V}}_{O_{2}\mathrm{peak}}$ and peak power (*W*_peak_). Participants were asked to indicate their RPE on the Borg scale at the start and end of each ${\dot{V}}_{O_{2}\mathrm{peak}}$ test. Indirect calorimetry (COSMED, Quark CPET; Rome, Italy) was used to collect and analyse expired gases, and ${\dot{V}}_{O_{2}\mathrm{peak}}$ was calculated from the highest 30 s average.

Participants performed a double‐leg, incremental ramp test to exhaustion on an electronically braked cycle ergometer (Lode, Excalibur Sport, v. 2.0; Groningen, Netherlands). Following a 1‐min warm‐up at 50 W, the workload was increased by 1 W every 2 s until the participant attained volitional exhaustion or cadence fell below 60 rpm. Participants performed a 5‐min cool‐down phase at 20 W, followed by 10 min of passive recovery.

Participants were familiarised with single‐leg cycling, as previously described (MacInnis, Zacharewicz et al., 2017), using a modified electronically braked cycle ergometer (Velotron, RacerMate; Seattle, WA, USA) modelled after previous work (Abbiss et al., 2011; Burns et al., 2014). Briefly, one crank on the ergometer was fitted with a custom‐machined pedal that held an 11.4 kg counterweight. Participants pedalled using the INT leg while the CON leg rested on a stationary platform. The counterweight assisted with the upstroke phase of the revolution, eliminating the need to pull upwards upon the pedal. The unilateral ${\dot{V}}_{O_{2}\mathrm{peak}}$ protocol mirrored the standard protocol except that the ramp increase was 1 W every 4 s. The two single‐leg tests were separated by 10 min since evidence indicates that limb fatigue is non‐transferable (Elmer et al., 2012).

#### 2.5.2 Training intervention

The training intervention consisted of a total of 12 INT sessions performed twice weekly for 6 weeks, and modelled on procedures previously described (MacInnis, Zacharewicz et al., 2017). Briefly, participants cycled at a cadence of 60–90 rpm and performed a 5‐min warmup at 25 W followed by 4 × 5‐min cycling bouts at an individualised workload that corresponded to 50%–60% *W*_peak_, interspersed with 2.5 min of recovery at 20% *W*_peak_. This intensity was selected since it constituted the approximate upper limit of tolerability for participants to sustain the 4 × 5‐min cycling bouts. Training progression increased by 5% *W*_peak_ every 2 weeks until the maximum workload of 60% *W*_peak_ was attained. The INT workload was based on the average single‐leg *W*_peak_ attained during the unilateral ${\dot{V}}_{O_{2}\mathrm{peak}}$ tests. Participants were asked to indicate their RPE on the Borg scale during the last 10 s of the interval and recovery sessions (e.g., 4 min 50 s, 8 min 50 s, etc.).

To contextualise the assigned training workloads, the gas exchange threshold (GET) and respiratory compensation point (RCP) were retrospectively determined for each participant from the single‐leg ${\dot{V}}_{O_{2}\mathrm{peak}}$ tests conducted at baseline on the leg assigned to INT. This enabled the determination of moderate (i.e., below GET), heavy (i.e., between GET and RCP) and severe intensity (i.e., above RCP) exercise domains for each participant. The GET and RCP were identified from the ramp ventilatory data by two experienced blinded investigators independently, using individual profiles of minute ventilation (${\dot{V}}_{E}$), volume of oxygen (${\dot{V}}_{O_{2}}$), volume of carbon dioxide (${\dot{V}}_{CO_{2}}$), partial pressure of end‐tidal oxygen ($P_{\mathrm{ET}O_{2}}$), partial pressure of end‐tidal carbon dioxide ($P_{\mathrm{ETC}O_{2}}$), ${\dot{V}}_{E}/{\dot{V}}_{O_{2}}$ and ${\dot{V}}_{E}/{\dot{V}}_{CO_{2}}$ plotted against ${\dot{V}}_{O_{2}}$. The GET was determined as the ${\dot{V}}_{O_{2}}$ at which ${\dot{V}}_{CO_{2}}$ and ${\dot{V}}_{E}$ begin to increase disproportionately relative to ${\dot{V}}_{O_{2}}$, with a systematic rise in $P_{\mathrm{ET}O_{2}}$ while $P_{\mathrm{ETC}O_{2}}$ and ${\dot{V}}_{E}/{\dot{V}}_{CO_{2}}$ remain stable (Iannetta, Inglis, Pogliaghi et al., 2020). The RCP was identified as the ${\dot{V}}_{O_{2}}$ at which $P_{\mathrm{ETC}O_{2}}$ began to fall following a period of isocapnia, coinciding with the second breakpoint in the ${\dot{V}}_{E}$–${\dot{V}}_{O_{2}}$ and ${\dot{V}}_{E}/{\dot{V}}_{CO_{2}}$–${\dot{V}}_{O_{2}}$ relationships (Iannetta, Inglis, Pogliaghi et al., 2020). When the two investigators' estimates differed by ≤100 mL min^−^^1^ they were averaged; when differences exceeded this threshold, a third investigator adjudicated.

### 2.6 Muscle biopsies

Muscle samples (∼100 mg) were obtained from the vastus lateralis of both legs before and after training under local anaesthesia (1% xylocaine) using the Bergström percutaneous needle biopsy technique (Tarnopolsky et al., 2011). Visible blood, connective tissue and adipose tissue were removed from the muscle, which was then dissected into two portions. Approximately half (∼50 mg) of the biopsy sample was oriented then embedded in optimal cutting temperature medium and frozen by immersion in isopentane, pre‐cooled in liquid nitrogen for immunofluorescence analyses. The remaining muscle was immediately flash‐frozen as described above. All samples were stored at −80°C for subsequent analyses.

### 2.7 Mitochondrial content

Mitochondrial content was assessed using a CS maximal activity assay and protein detection by western immunoblotting. Muscle samples (∼25 mg) were homogenised in 20 volumes of buffer in a tube with one 3.2 mm steel bead. The tubes were run through 2 × 40 s cycles at a speed of 20 shakes/s on the Qiagen TissueLyser (Retsch, 85210; Haan, Germany) and placed on ice for 5 min between cycles. Samples were centrifuged at 12,000 *g* for 10 min at 4°C, and then the homogenate supernatant was collected and stored at −80°C for future experiments. Homogenates were assessed for total protein concentration using a bicinchoninic acid assay according to manufacturer's instructions. All antibodies used for experiments are presented in Table 2.

**TABLE 2:** Antibodies and dye.

Antibodies	Isotype	Species	Dilution	Stock concentration	Company and cat. no.	RRID
Primary antibodies						
Anti‐CD31	IgG	Rabbit	1:25	0.013 mg mL^−^^1^	Abcam, ab28364	AB_726362
Anti‐Laminin (β‐2/γ‐1, A5)	IgG2a	Rat	1:200	1 mg mL^−^^1^	Thermo Fisher Scientific, MA1‐06100	AB_559896
Anti‐MHC I ^a^	IgG2b	Mouse	1:100	0.024 mg mL^−^^1^	DSHB, BA‐D5	AB_2235587
Total OXPHOS Human Western Blot Antibody Cocktail	IgG	Mouse	1:500	1.5 mg mL^−^^1^	Abcam, ab110411	AB_2756818
Secondary antibodies						
AF488 goat, anti‐rabbit	IgG	Goat	1:100	2 mg mL^−^^1^	Thermo Fisher Scientific, A‐11034	AB_2576217
AF594 goat, anti‐rat	IgG	Goat	1:300	2 mg mL^−^^1^	Thermo Fisher Scientific, A‐11007	AB_10561522
AF647 goat, anti‐mouse^b^	IgG, Fcγ2b	Goat	1:100	0.8 mg mL^−^^1^	Jackson ImmunoResearch Laboratories, 115‐605‐207	AB_2338918
Anti‐mouse, HRP	IgG	Horse	1:1000	0.153 mg mL^−^^1^	Cell Signaling Technology, 7076	AB_330924
Dye						
DAPI	—	—	1:20,000	20 mg mL^−^^1^	Sigma‐Aldrich, D9542	—

*Note*: Antibodies and nuclear dye used for experiments.

^a^ Deposited to the DSHB by S. Shiaffino.

^b^ AF647 initial (1.6 mg mL^−1^) aliquoted 1:1 in sterile glycerol. Abcam, Waltham, MA, USA; Cell Signaling Technology, Danvers, MA, USA; Jackson ImmunoResearch Laboratories, West Grove, PA, USA; Sigma‐Aldrich, St Louis, MO, USA; Thermo Fisher Scientific, Waltham, MA, USA.

Abbreviations: AF, Alexa Fluor; Cat. No., manufacturer's catalog product number; CD31, cluster of differentiation 31; DAPI, 4′,6‐diamidino‐2‐phenylindole; DSHB, Developmental Studies Hybridoma Bank; HRP, horseradish peroxidase; MHC, myosin heavy chain type I; RRID, research resource identifier.

#### 2.7.1 Citrate synthase maximal activity

All products used for CS assays are presented in Appendix Table A1. CS maximal activity was assessed using previously published protocols with modifications to fit a 96‐well plate (Gianni et al., 2004). Samples were homogenised in buffer containing 70 mM sucrose, 220 mM mannitol, 10 mM HEPES, 1 mM EGTA, and supplemented with protease inhibitors. CS activity was measured by adding 4.5 µl of muscle homogenate to wells containing: 247.5 µl 0.1 M Tris‐hydrochloride (pH 8.0), 30 µl 5,5′‐dithiobis (2‐nitrobenzoic acid) (0.5 mg mL^−1^ Tris buffer) and 3 µl acetyl CoA (6 mg mL^−1^ Tris‐hydrochloride). The plate was warmed for 5 min at 37°C, then 50 µL of oxaloacetate (6.1 mg mL^−1^ Tris buffer) was added to initiate the reaction. Absorbance was recorded at 412 nm for 120 s on a Synergy2 plate reader (BioTek; Winooski, VT, USA). The slope between 30 and 90 s was used to calculate the reaction rate, and CS maximal activity was determined as previously described (Janssen & Boyle, 2019). The extinction coefficient selected for the reaction of 5‐thio‐2‐nitrobenzoic acid was 14,150 M cm^−1^ (Riddles et al., 1983). Duplicates for each sample were compared and final enzyme activities were normalised to total protein content.

### 2.8 Western immunoblotting

All products used for western immunoblotting are presented in Appendix Table A2. Mitochondrial complex protein contents were assessed using the western immunoblotting technique, as previously described, with modifications (Skelly et al., 2023). Briefly, samples were homogenised in radioimmunoprecipitation assay buffer supplemented with protease and phosphatase inhibitors and stored at −80°C. Homogenised samples were mixed into loading buffer to a concentration of 2 µg µL^−1^, and 20 µg of protein was loaded per lane in the gel. A protein ladder and a standard calibration curve of pooled samples were run on each gel.

Prepared samples were heated to 37°C for 5 min then loaded into a 4%–20% precast gel and run for 45 min at 200 V at room temperature (∼21°C). The gel was transferred to a membrane and verified using Ponceau stain. Membranes were washed 3 × 5 min with 1 × Tris‐buffered saline with 0.1% Tween (1× TBST), then blocked for 1 h in 5% milk with 1× TBST, then incubated in the total OXPHOS primary antibody cocktail in 5% bovine serum albumin overnight at 4°C. The following day membranes were washed and incubated for 1 h at room temperature in the anti‐rabbit secondary antibody in 5% milk. Membranes were incubated for 5 min in (1:1) Super Signal Pico PLUS chemiluminescent substrate (Thermo Fisher Scientific, 34580; Waltham, MA, USA) and imaged. All imaging was performed using a ChemiDoc MP imaging system (Bio‐Rad Laboratories; Hercules, CA, USA). The blots were quantified using Image Lab (Bio‐Rad, v. 6.1). After background correction of the bands the protein contents were expressed relative to the standard calibration curve and normalised to total protein content. Representative western immunoblot images are presented in Figure 3.

**FIGURE 3:**
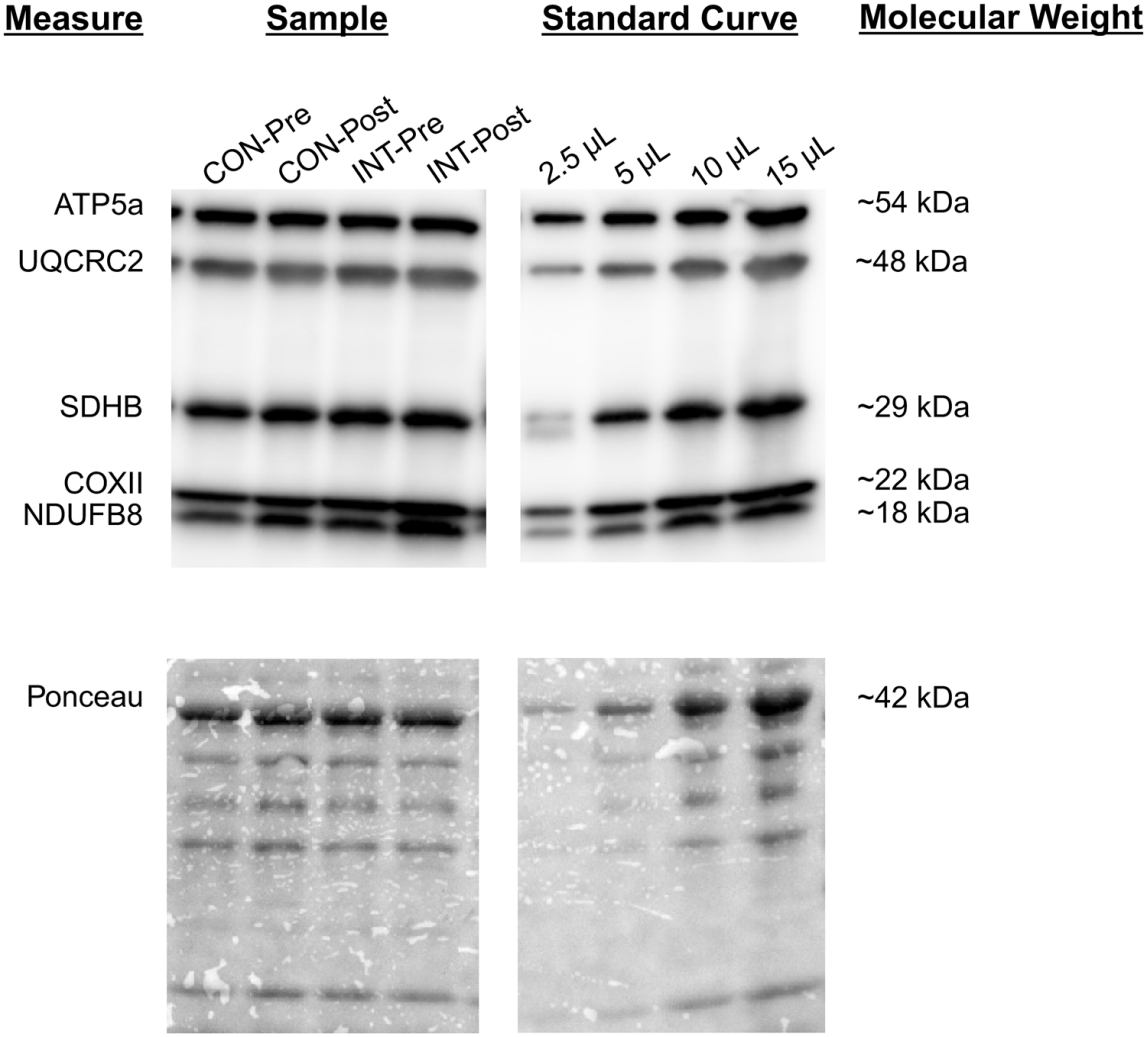
Representative image of western immunoblotting. All timepoints for individual participant samples were run on the same gel (e.g., Participant no. 1: CON‐Pre, CON‐Post, INT‐Pre, and INT‐Post). The standard calibration curve consisted of pooled whole‐muscle samples loaded in different volumes for different amounts of protein (i.e., 5, 10, 20, and 30 µg). Created in BioRender. Gibala, M. (2026) https://BioRender.com/ep4nbej.

### 2.9 Immunofluorescence

All antibodies and products used for immunofluorescence are presented in Appendix Table A3. Samples were cryo‐sectioned (Leica, CM 1950; Wetzler, Germany) to a thickness of 10 µm and then stored at −80°C until immunofluorescence staining. Unless otherwise stated, staining was conducted at room temperature (∼21°C) in the dark. All antibodies and blocking mixtures were formulated with 1× phosphate buffered saline while all washing steps were conducted using 1× TBST for a total of 3 × 5 min. To confirm secondary antibody binding specificity, the primary antibodies were omitted from three muscle sections. Prior to staining all samples were air dried at room temperature (∼21°C) for 15 min and staining margins were outlined with an ImmEdge PAP pen (Vector Laboratories; Newark, CA, USA). Immunofluorescent staining was conducted for myosin heavy chain isoform identification and capillarisation, as previously described, with modifications (Binet et al., 2023).

Briefly, sections were fixed in 4% paraformaldehyde for 5 min, washed, blocked for 1 h in 5% fetal bovine serum, 5% goat serum, 0.2% Triton X‐100, then incubated overnight at 4°C in primary antibody anti‐CD31 in 5% goat serum, 0.2% Triton X‐100. The following day, sections were washed and then incubated for 2 h in the secondary antibody AF488 in 5% goat serum. Sections were washed, blocked for 1 h in 5% bovine serum albumin, then incubated for 4 h in the following primary antibody mixture: BA‐D5 and laminin in 1% bovine serum albumin, 0.5% Triton X‐100. Sections were washed then incubated for 2 h in the following secondary antibody mixture: AF594 (laminin) and AF647 (BA‐D5) in 0.5% bovine serum albumin, 5% goat serum. Sections were washed, incubated in 4',6‐diamidino‐2‐phenylindole for 5 min, washed for 5 min, then mounted and cover‐slipped. Mounted slides were stored at 4°C until visualisation using widefield fluorescence microscopy. A representative immunofluorescence image is presented in Figure 4.

**FIGURE 4:**
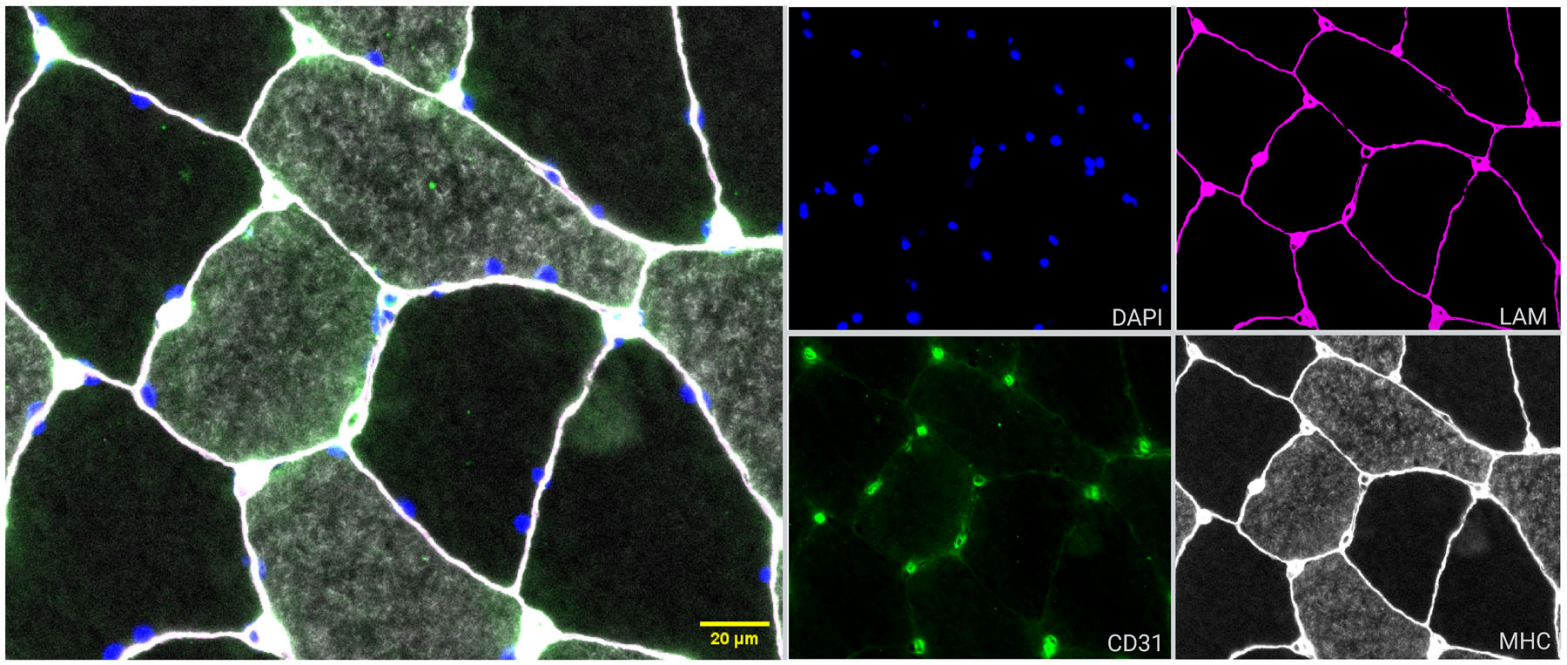
Representative image of immunofluorescence. Image of capillaries captured at ×20, 0.80 NA. Type I (BA‐D5^+^) fibres are shown in grey, while type II (BA‐D5^–^) fibres appear unstained. CD31, cluster of differentiation 31; DAPI, 4',6‐diamidino‐2‐phenylindole; LAM, laminin; MHC, myosin heavy chain. Created in BioRender. Gibala, M. (2026) https://BioRender.com/wg2ecxn.

#### 2.9.1 Muscle fibre capillarisation imaging and analysis

Images were captured at ×20, 0.80 NA, plan apochromat M27 objective using an AxioCam 712 monochromatic camera with pixel size of 3.45 µm, on a ZeissAxioscan 7 (Oberkochen, Germany) wide‐field fluorescence microscope (RRID: SCR_020927). The following LEDs were used on the Colibri 7 light‐source: 385, 475, 5967 and 630 nm. Images were acquired using the same parameters at a resolution of 4096 × 3008 pixels, then converted to an 8‐bit tiff. All images were adjusted to the same brightness and contrast, then analysed using Fiji/ImageJ (National Institutes of Health, v. 1.53t; Bethesda, MD, USA).

Fibre type was examined by myosin heavy chain isoforms and was identified as follows: type I, BA‐D5^+^; type II, BA‐D5^–^. The following capillarisation measures were recorded for each individual fibre analysed, as previously described (Hepple et al., 1997): (1) capillary contacts (CC), defined as the number of capillaries surrounding each fibre; (2) capillary density (CD), based on cross‐sectional area; (3) capillary‐to‐fibre ratio per individual fibre (C/F_i_); and (4) capillary‐to‐fibre perimeter exchange (CFPE) index. Fibre type proportion along with capillarisation was expressed as a ratio of fibre type expressing each myosin heavy chain isoform, to the total number of fibres analysed. All intact fibres were analysed, whereas longitudinally cut regions, folded, overlapping, damaged or incomplete sections were excluded. It has previously been recommended that a minimum of 50 fibres (i.e., 25 type I and 25 type II) be analysed to quantify muscle fibre capillarisation (Porter et al., 2002). Thus, we measured a mean ± standard deviation (SD) of 127 ± 57 (type I: 54 ± 32, type II: 75 ± 42) fibres per section. All image analyses were performed manually, by the same investigator blinded to the experimental condition.

### 2.10 Statistical analyses

Statistical analyses were conducted and figures were constructed using GraphPad Prism software (v. 10.6.1; GraphPad Software, Boston, MA, USA). Data were tested for a normal distribution using the Shapiro–Wilk normality test, and abnormal data were log transformed (*y* = log(*y*)). Data were analysed using a two‐factor (time × group), within‐subject, repeated measures ANOVA. The time factor constitutes pre‐training versus post‐training, while the group factor constitutes CON versus INT conditions. Significant interactions were assessed using the Bonferroni multiple‐comparisons *post hoc* test. Abnormal data were assessed using the non‐parametric Friedman's test. Data are expressed as means ± SD with significance set at *P* ≤ 0.05. Effect sizes are reported as partial eta‐squared (η_p_^2^) and expressed as small (0.01), medium (0.06), or large (0.14) (Lakens, 2013). Effect sizes are not reported for non‐parametric data or data analysed using a mixed‐effects model. The 95% confidence intervals (CI) are presented as upper and lower limits. Measures of variability between the CON and INT legs at baseline were expressed as a relative typical error percentage (TE) as previously described (Bostad et al., 2023; Hopkins, 2000). Briefly, the change scores were calculated from the subtraction of the CON leg value from the INT leg value at baseline, and the SD was calculated from the change scores. The grand mean was the mean of all values included in the TE calculation. Pearson correlation coefficients were examined to assess the relationships between the change in percentage GET and percentage RCP compared to measures of mitochondrial content and capillarisation in the INT leg.

## 3 RESULTS

### 3.1 Exercise training data

Mean power output during the 5‐min work bouts of the INT sessions was 67 ± 14, 73 ± 16 and 80 ± 17 W during weeks 1–2, 3–4 and 5–6, respectively, with an overall mean training workload of 73 ± 16 W. Mean heart rate during the work bouts of the INT sessions was 122 ± 17 (CI: 113, 131) beats min^−1^.

Mean RPE (6–20 Borg Scale) during the bouts were 13 ± 2 (CI: 12, 14) arbitrary units (AU).

The GET and RCP, retrospectively determined from the single‐leg ${\dot{V}}_{O_{2}\mathrm{peak}}$ tests conducted at baseline on the INT leg, corresponded to power outputs of 72 ± 21 (CI: 61, 82) and 122 ± 31 (CI: 107, 137) W, respectively. Expressed relative to baseline single‐leg *W*_peak_, the power outputs at GET and RCP constituted 54 ± 10 (CI: 49, 59)% and 90 ± 8 (CI: 86, 97)%, respectively, of baseline single‐leg *W*_peak_. Mean power output during the 5‐min work bouts over the entire 6‐week intervention corresponded to 105 ± 18 (CI: 96, 114) % GET and 61 ± 7 (CI: 58, 65) % RCP. Mean training intensity was therefore in the heavy intensity domain (i.e., above GET but below RCP). This varied, however, depending on the participant and stage of the intervention. During the first two weeks of training, eight participants trained in the moderate domain and eight in the heavy domain. The workload progression was such that, during the last two weeks of training, five participants still worked in the moderate domain and 11 trained in the heavy domain. No participants trained in the severe intensity domain at any point in the training intervention.

### 3.2 Single‐leg ${\dot{V}}_{O_{2}\mathrm{peak}}$ and *W*_peak_

Single‐leg ${\dot{V}}_{O_{2}\mathrm{peak}}$ showed a main effect of time (*P* < 0.001, Ƞ_p_^2^ = 0.394) such that it was higher after the intervention compared to before training in both the CON and INT legs (Figure 5a). ${\dot{V}}_{O_{2}\mathrm{peak}}$ in the CON leg was 27.5 ± 5.0 (CI: 24.7, 30.3) and 30.1 ± 4.9 (CI: 27.4, 32.8) mL kg^−1^ min^−1^ before and after the intervention, respectively. ${\dot{V}}_{O_{2}\mathrm{peak}}$ in the INT leg was 28.4 ± 4.1 (CI: 26.2, 30.7) and 31.0 ± 5.3 (CI: 28.1, 33.9) mL kg^−1^ min^−1^ before and after the intervention, respectively. The TE for ${\dot{V}}_{O_{2}\mathrm{peak}}$ between both legs at baseline was 8.2%.

**FIGURE 5:**
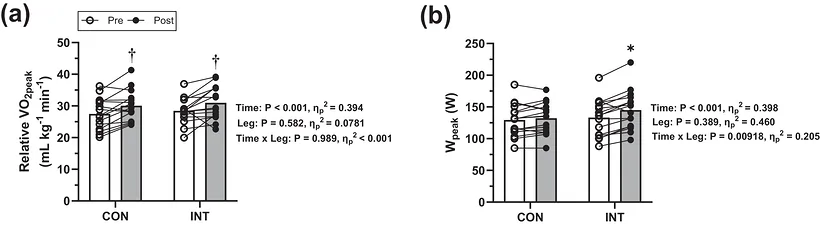
Single‐leg ${\dot{V}}_{O_{2}\mathrm{peak}}$ and *W*_peak_. Mean and individual participant data are presented for measurements made before (Pre) and after (Post) the intervention in each of the Control (CON) and Interval (INT) leg. (a) Single‐leg relative ${\dot{V}}_{O_{2}\mathrm{peak}}$ (*n* = 15; 6 males, 9 females). Data were analysed using a mixed‐effects model. †Significant effect of time (*P* < 0.001). (b) Single‐leg *W*_peak_ (*n* = 16). Data were analysed using a two‐factor, repeated measures ANOVA with a Bonferroni *post hoc* test. *Significant time × leg interaction (*P* = 0.00918).

Single‐leg *W*_peak_ showed a significant time × leg interaction (*P* = 0.00918, Ƞ_p_^2^ = 0.21) such that it was higher after the intervention compared to before training in the INT leg (*P* < 0.001, Figure 5b). *W*_peak_ in the CON leg was 129 ± 26 (CI: 116, 143) and 132 ± 24 (CI: 119, 145) W before and after the intervention, respectively. *W*_peak_ in the INT leg was 133 ± 29 (CI: 118, 149) and 145 ± 31 (CI: 129, 161) W before and after the intervention, respectively. The TE for *W*_peak_ between both legs at baseline was 5.9%.

### 3.3 CS maximal activity

There were no differences in the primary outcome measure of CS maximal activity within or between legs at any time point (Figure 6a). CS maximal activity in the CON leg was 64.4 ± 21.1 (CI: 53.2, 75.7) and 62.8 ± 20.0 (CI: 52.2, 73.4) nmol min^−1^ mg protein^−1^ before and after the intervention, respectively. CS maximal activity in INT leg was 73.7 ± 28.6 (CI: 58.4, 89.0) and 80.8 ± 25.1 (CI: 67.4, 94.1) nmol min^−1^ mg protein^−1^ before and after training, respectively. The TE for CS between both legs at baseline was 34.5%.

**FIGURE 6:**
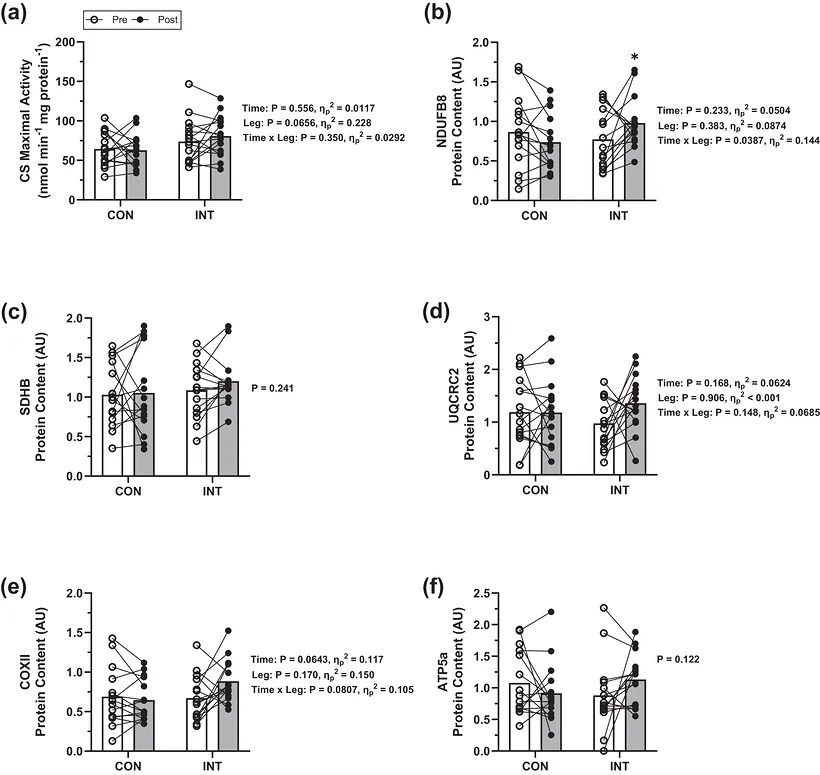
Biomarkers of mitochondrial content. Mean and individual participant data are presented for measurements made before (Pre) and after (Post) the intervention in each of the Control (CON) and Interval (INT) leg. (a) Citrate synthase maximal activity (*n* = 16). (b) NDUFB8 (*n* = 15; 6 males, 9 females). (c) SDHB (*n* = 15; 5 males, 10 females). (d) UQCRC2 (*n* = 16). (e) COXII (*n* = 15; 5 males, 10 females). (f) ATP5a (*n* = 15; 6 males, 9 females). Data in panels (a, b, d, e) were analysed using a two‐factor, repeated measures ANOVA with a Bonferroni *post hoc* test. Data in panels (c, f) were analysed using the non‐parametric Friedman's test. *Significant time × leg interaction (*P* = 0.0387).

There was no correlation between the change in CS versus the change in ${\dot{V}}_{O_{2}\mathrm{peak}}$ and the change in CS versus the change in *W*_peak_ in the INT leg (all *P* > 0.05, data not shown). There was also no correlation between the change in CS compared to training intensity expressed as % GET and % RCP the INT leg (*P* > 0.05, data not shown).

### 3.4 Mitochondrial protein content

There were no differences in most measures of mitochondrial protein content. The exception was complex I biomarker NDUFB8 (Figure 6b), which showed a time × leg interaction (*P* = 0.0387, Ƞ_p_^2^ = 0.144) such that it increased in the INT leg (*P* = 0.0470). NDUFB8 protein content in the CON leg was 0.87 ± 0.46 (CI: 0.62, 1.12) and 0.74 ± 0.35 (CI: 0.55, 0.93) AU before and after training, respectively. NDUFB8 protein content in the INT leg was 0.77 ± 0.37 (CI: 0.57, 0.98) and 0.98 ± 0.32 (CI: 0.80, 1.16) AU before and after training, respectively. The TE for NDUFB8 protein content between both legs at baseline was 39.1%. There was no correlation between the change in NDUFB8 protein content compared to training intensity expressed as % GET and % RCP in the INT leg (*P* > 0.05, data not shown).

There were no differences in complex II biomarker SDHB protein content (Figure 6c). SDHB protein content in the CON leg was 1.03 ± 0.39 (CI: 0.81, 1.25) and 1.05 ± 0.53 (CI: 0.76, 1.35) AU before and after training, respectively. SDHB protein content in the INT leg was 1.09 ± 0.35 (CI: 0.89, 1.28) and 1.20 ± 0.31 (CI: 1.03, 1.37) AU before and after training, respectively. The TE for SDHB protein content between both legs at baseline was 31.8%. There was no correlation between the change in SDHB protein content compared to training intensity expressed as % GET and % RCP in the INT leg (*P* > 0.05, data not shown).

There were no differences in complex III biomarker UQCRC2 protein content (Figure 6d). UQCRC2 protein content in the CON leg was 1.19 ± 0.65 (CI: 0.84, 1.54) and 1.18 ± 0.63 (CI: 0.85, 1.51) AU before and after training, respectively. UQCRC2 protein content in the INT leg was 0.98 ± 0.47 (CI: 0.72, 1.23) and 1.36 ± 0.51 (CI: 1.09, 1.63) AU before and after training, respectively. The TE for UQCRC2 protein content between both legs at baseline was 45.4%. There was no correlation between the change in UQCRC2 protein content compared to training intensity expressed as % GET and % RCP in the INT leg (*P* > 0.05, data not shown).

There were no differences in complex IV biomarker COXII protein content (Figure 6e). COXII protein content in the CON leg was 0.69 ± 0.36 (CI: 0.49, 0.89) and 0.65 ± 0.26 (CI: 0.50, 0.79) AU before and after training, respectively. COXII protein content in the INT leg was 0.67 ± 0.29 (CI: 0.51, 0.83) and 0.88 ± 0.27 (CI: 0.75, 1.0) AU before and after training, respectively. The TE for COXII protein content between both legs at baseline was 37.4%. There was no correlation between the change in COXII protein content in the INT leg compared to training intensity expressed as % GET; however, there was a low, positive correlation (*R* ^2^ = 0.267) when compared to % RCP (*P* > 0.0487, data not shown).

There were no differences in complex IV biomarker ATP5a protein content (Figure 6f). ATP5a protein content in the CON leg was 1.08 ± 0.52 (CI: 0.79, 1.36) and 0.91 ± 0.48 (CI: 0.65, 1.18) AU before and after training, respectively. ATP5a protein content in the INT leg was 0.88 ± 0.57 (CI: 0.57, 1.20) and 1.13 ± 0.41 (CI: 0.91, 1.36) AU before and after training, respectively. The TE for ATP5a protein content between both legs at baseline was 45.8%. There was no correlation between the change in ATP5a protein content compared to training intensity expressed as % GET and % RCP in the INT leg (*P* > 0.05, data not shown).

### 3.5 Fibre capillarisation

Fibre characteristics and capillarisation measures are presented in Tables 3 and 4, respectively. There was no time × leg interaction and no main effects of time and leg, detected for all fibre characteristics (Table 3, all *P* > 0.05). A significant time × leg interaction (*P* = 0.0333, Ƞ_p_^2^ = 0.163) was observed for type II fibre CC (Table 4). There was no time × leg interaction and no main effects of time and leg, detected for the remaining measures of capillarisation: type I fibre CC and CD, C/Fi, and CFPE for both fibre types (Table 4, all *P* > 0.05). The TE between both legs at baseline for measures of capillarisation were as follows: CC, TE: I = 19.5%, II = 17.3%; C/Fi, TE: I = 20.7%, II = 18.2%; CD, TE: I = 14.0%, II = 14.6%; and CFPE index, TE: I = 17.6%, II = 18.2%. There were no correlations between the change in all measures of capillarisation compared to training intensity expressed as % GET and % RCP in the INT leg (*P* > 0.05, data not shown).

**TABLE 3:** Fibre characteristics.

Measure	CON‐Pre	CON‐Post	INT‐Pre	INT‐Post	*P* [ηp^2^]		
					Time	Leg	Interaction
Cross‐sectional area‐I (µm^2^)	4089 ± 1295 (3341, 4836)	4201 ± 752 (3768, 4636)	3889 ± 1123 (3241, 4537)	4153 ± 919 (3622, 4684)	0.151 [0.0776]	0.668 [0.025]	0.735 [0.00449]
Cross‐sectional area‐II (µm^2^)	3793 ± 955 (3242, 4344)	4182 ± 698 (3779, 4585)	4163 ± 1104 (3526, 4801)	4114 ± 959 (3560, 4667)	0.290 [0.0429]	0.684 [0.0161]	0.210 [0.0598]
Myonuclei‐I	2.52 ± 0.34 (2.33, 2.72)	2.69 ± 0.48 (2.41, 2.97)	2.63 ± 0.54 (2.31, 2.94)	2.54 ± 0.42 (2.30, 2.78)	0.747 [0.00409]	0.876 [< 0.001]	0.331 [0.0364]
Myonuclei‐II	2.52 ± 0.24 (2.38, 2.66)	2.76 ± 0.57 (2.43, 3.09)	2.80 ± 0.52 (2.50, 3.11)	2.72 ± 0.57 (2.39, 3.05)	0.448 [0.0223]	0.430 [0.0525]	0.135 [0.0838]
Myonuclear domain‐I (µm^2^)	1960 ± 478 (1684, 2236)	1919 ± 314 (1737, 2100)	1843 ± 309 (1665, 2021)	1912 ± 437 (1660, 2165)	0.877 [< 0.001]	0.607 [0.0185]	0.535 [0.0150]
Myonuclear domain‐II (µm^2^)	1817 ± 495 (1532, 2103)	1883 ± 321 (1698, 2068)	1790 ± 240 (1652, 1929)	1839 ± 414 (1600, 2078)	0.519 [0.0162]	0.827 [0.00265]	0.692 [0.00614]
Fibre proportion I (%)	47 ± 15 (39, 56)	40 ± 11 (34, 46)	44 ± 20 (33, 55)	45 ± 15 (36, 54)	0.189 [0.0655]	0.886 [0.00355]	0.113 [0.0941]
Fibre proportion II (%)	53 ± 15 (44, 61)	60 ± 11 (54, 67)	56 ± 20 (45, 67)	55 ± 15 (46, 64)	—	—	—

*Note*: Data (*n* = 14; 4 males 10 females) presented as means ± SD (95% confidence interval lower‐ and upper‐bounds). Data were analysed using a two‐factor, repeated measures ANOVA. Fibre type proportion *P*‐values and effect sizes were equivalent and are presented as combined values.

Abbreviations: ηp^2^, partial eta squared; I, type I fibres; II, type II fibres.

**TABLE 4:** Fibre capillarisation.

Measure	CON‐Pre	CON‐Post	INT‐Pre	INT‐Post	*P* [ηp^2^]		
					Time	Leg	Interaction
CC‐I (AU)	1.95 ± 0.39 (1.73, 2.18)	2.17 ± 0.35 (1.97, 2.37)	2.16 ± 0.65 (1.79, 2.54)	1.86 ± 0.48 (1.59, 2.14)	0.752 [0.00392]	0.704 [0.00433]	0.0717 [0.119]
CC‐II (AU)	1.79 ± 0.28 (1.63, 1.95)	1.98 ± 0.32 (1.80, 2.16)	2.00 ± 0.47 (1.73, 2.27)	1.71 ± 0.47 (1.44, 1.98)	0.649 [0.00809]	0.761 [0.00366]	0.0333^*^ [0.163]
C/Fi‐I (AU)	0.70 ± 0.16 (0.61, 0.79)	0.78 ± 0.16 (0.69, 0.87)	0.78 ± 0.24 (0.64, 0.92)	0.69 ± 0.20 (0.58, 0.81)	0.994 [< 0.001]	0.905 [< 0.001]	0.106 [0.0975]
C/Fi‐II (AU)	0.64 ± 0.11 (0.57, 0.71)	0.70 ± 0.13 (0.62, 0.78)	0.70 ± 0.17 (0.60, 0.80)	0.61 ± 0.18 (0.51, 0.72)	0.702 [0.00575]	0.795 [0.00299]	0.0661 [0.124]
CD‐I (no. cap. mm^−^^2^)	520 ± 96 (465, 576)	545 ± 136 (467, 624)	611 ± 154 (522, 700)	489 ± 177 (387, 591)	0.197 [0.0633]	0.670 [0.00871]	0.0541 [0.135]
CD‐II (no. cap. mm^−^^2^)	504 ± 124 (433, 576)	484 ± 129 (410, 558)	502 ± 88 (451, 553)	441 ± 133 (364, 518)	0.896	—	—
CFPE Index‐I (no. cap. 1000 µm^−^^1^)	2.80 ± 0.42 (2.56, 3.04)	3.01 ± 0.56 (2.69, 3.33)	3.19 ± 0.83 (2.72, 3.67)	2.75 ± 0.77 (2.31, 3.20)	0.545 [0.0143]	0.677 [0.00540]	0.0917 [0.105]
CFPE Index‐II (no. cap. 1000 µm^−^^1^)	2.59 ± 0.34 (2.39, 2.79)	2.64 ± 0.60 (2.30, 2.99)	2.78 ± 0.52 (2.48, 3.08)	2.41 ± 0.66 (2.04, 2.79)	0.278 [0.0451]	0.889 [< 0.001]	0.153 [0.0769]

*Note*: Data (*n* = 14; 4 males 10 females) presented as mean ± SD (95% confidence interval lower‐ and upper‐bounds). Data were analysed using a two‐factor, repeated measures ANOVA, with the exception of CD‐II which was analysed using the non‐parametric Friedman's test.

* Significant time × leg interaction.

Abbreviations: ηp^2^, partial eta squared; I, type I fibres; II, type II fibres; AU, arbitrary units; CC, capillary contacts; CD, capillary density; C/Fi, capillary to fibre ratio; CFPE, capillary‐to‐fibre perimeter exchange; no. cap., number of capillaries.

## 4 DISCUSSION

The primary novel finding from the present study was that biomarkers of skeletal muscle oxidative capacity were unchanged after twice‐weekly INT performed for 6 weeks. The design incorporated a single‐leg, counterweighted cycling model, facilitating a within‐participant design and comparison of training responses with a control leg that did not train. There was evidence of a training effect in that single‐leg *W*_peak_ increased in the INT leg compared to pre‐training, and the CON leg. However, mitochondrial content was largely unchanged in both legs as evidenced by no differences in our primary outcome, CS maximal activity, and the protein content of four of five mitochondrial complex biomarkers. Fibre capillarisation as measured by CC, CD, C/Fi and CFPE index also remained unchanged in both legs.

### 4.1 Mitochondrial content

To our knowledge, the present study is the first to examine the effect of twice‐weekly INT on skeletal muscle oxidative capacity using a single‐leg training design. Our primary outcome CS maximal activity and the five other mitochondrial proteins measured are common biomarkers that correlate with mitochondrial content as determined by transmission electron microscopy (Larsen et al., 2012). In contrast to our hypothesis, we observed no change in CS maximal activity and four of the five protein content markers. The exception was NDUFB8 protein content, a biomarker of mitochondrial complex I, which increased in the INT leg following training

The present findings contrast with a previous study from our laboratory (MacInnis, Zacharewicz et al., 2017) that reported increased skeletal muscle mitochondrial content after six sessions of INT over 2 weeks in young, active adults. That study used the same single‐leg, counterweighted cycling model and employed an INT intervention and a relative training workload similar to those in the present study, except that three weekly sessions were performed. The previous study did not make comparisons to a non‐exercise control, but rather the contralateral limb performed an equivalent number of continuous training sessions at a lower intensity that was matched for total work and time (MacInnis, Zacharewicz et al., 2017). The study showed a training‐induced increase in CS maximal activity that was greater than in the leg that performed continuous training and increases in specific mitochondrial complexes including COXIV, NDUFA9 and MFN2 that were similar between legs (MacInnis, Zacharewicz et al., 2017). The relative training intensity in our previous study (60%–65% *W*_peak_) was also slightly higher than in the present study (50%–60% *W*_peak_). Our original intent was to precisely match the relative intensity of the INT sessions with our previous study, but in familiarisation sessions our participants were only able to achieve a starting intensity of ∼50% *W*_peak_ for the 4 × 5‐min work bouts and this was gradually increased over the intervention to 60% *W*_peak_ during the final phase of training.

Another previous study from our laboratory used the single‐leg, counterweighted cycling model comparing two training interventions: INT versus continuous training. The INT leg performed 10 × 3‐min bouts at 50% *W*_peak_, while the contralateral limb performed continuous training lasting 30 min at 50% *W*_peak_ (Skelly et al., 2023). A biopsy was obtained from only one leg at baseline prior to initiation the training interventions followed by both legs after training, thus facilitating a one‐way comparison across the three conditions (i.e., baseline, INT, continuous). The study showed that after 4 weeks of training performed thrice weekly, there was no change in CS maximal activity after INT but an increase in the leg that performed continuous training (Skelly et al., 2023) compared to the pre‐training time point. There was also no difference in mitochondrial protein complex biomarkers after the INT intervention, which is largely consistent with the present work (Skelly et al., 2023). Overall, when investigated using a single‐leg training model that enables comparisons to a control group or continuous exercise training, the magnitude and time course of change in skeletal muscle mitochondrial content in response to several weeks of INT remains equivocal. This is in contrast to sprint interval studies of comparable duration that have used whole‐body ‘all‐out’ cycling models and reported increases in skeletal muscle mitochondrial content after thrice‐weekly training for several weeks (Gibala & MacInnis, 2022).

### 4.2 Fibre capillarisation

Changes in fibre capillarisation have been shown to occur relatively early on in training (i.e., <4 weeks) and appear to be primarily driven by higher training intensities and continuous training frequencies, with changes being most apparent in untrained adults (Mølmen et al., 2025; Rosenblat et al., 2022). We measured several markers of fibre capillarisation (CC, CD, C/Fi and CFPE) and they remained unchanged by INT. Similar results were observed in a study investigating the effects of 4 weeks of INT, preceded by 4 weeks of continuous training in young adults (Hoier et al., 2013). This study showed that fibre capillarisation, as measured by CD, and C/Fi only increased following continuous training and not INT (Hoier et al., 2013).

Conversely, most studies involving higher training intensities and frequencies observed changes in fibre capillarisation following INT including some studies that made comparisons with continuous training (Bonafiglia et al., 2017; Cocks et al., 2013, 2016; Daussin et al., 2008; Raleigh et al., 2018; Scott et al., 2019; Scribbans et al., 2014; Tan et al., 2018). For example, previous work in our laboratory showed that fibre capillarisation, as measured by CC, CD and C/Fi increased in older, overweight females following 6 weeks of INT (Tan et al., 2018). Similarly, capillarisation as measured by CC and CD were also increased following 4–6 weeks of INT and continuous training in young, healthy males (Cocks et al., 2013, 2016) with no difference between training types. Furthermore, higher training frequencies of four sessions per week showed increased CD following 4–6 weeks of INT (Bonafiglia et al., 2017; Scribbans et al., 2014) and 4 weeks of INT (Raleigh et al., 2018) in young, healthy adults. Longer duration training interventions of thrice‐weekly INT, lasting 12 weeks has also been shown to increase CC, CD, C/Fi and CFPE index (Scott et al., 2019).

### 4.3 Characterisation of training intensity

We modelled the current study on our previous work to make comparisons between interventions of a lower training frequency (MacInnis, Zacharewicz et al., 2017; Skelly et al., 2023). This entailed prescribing INT intensity as a percentage of *W*_peak_, a method that is known to elicit heterogeneous acute physiological responses to exercise due to the substantial inter‐individual variability in intensity domain boundaries (Iannetta, Inglis, Mattu et al., 2020). We also therefore retrospectively determined the power outputs corresponding to the GET and RCP for all participants during the baseline single‐leg cycling tests and assessed this relative to the mean power outputs during training. As the GET and RCP are common metabolic thresholds used to delineate exercise domains, this enabled a determination of whether each participant was working in the moderate, heavy or severe exercise domain during training and in turn an assessment of adaptive responses relative to intensity domain. The results of this retrospective analysis showed that participants were mainly training in the moderate or heavy exercise domain and thus below the severe intensity domain typically associated with high‐intensity interval training during more traditional whole‐body exercise (Coates et al., 2023). A limitation of our retrospective approach to determine exercise domains was that the use of a single graded exercise test to determine GET and RCP is challenging due to ventilatory kinetic lag resulting in an overestimated power output for each threshold (Keir et al., 2018). Use of a ‘Step‐Ramp‐Step’ protocol has been proposed as a practical method to accurately identify the corrected (i.e., lower) power output at these metabolic thresholds (Iannetta, Inglis, Pogliaghi et al., 2020). Compared with double‐leg cycling, single‐leg counter‐weighted cycling has also been shown to elicit higher heart rate, ventilation and oxygen uptake when expressed per unit workload (i.e., per watt), particularly at higher intensities (MacInnis, Morris et al., 2017). Our previous study also showed that single‐leg, counter‐weighted cycling at ∼60%–65% *W*_peak_ elicited relatively high venous blood lactate (>8 mmol), which is suggestive of relatively strenuous exercise (MacInnis, Zacharewicz et al., 2017).

### 4.4 Single leg peak oxygen uptake

Single‐leg ${\dot{V}}_{O_{2}\mathrm{peak}}$ showed a main effect, with higher values after the intervention than pre‐training. This could indicate an initial limitation in oxygen delivery and potential cardiovascular responses to training beyond the capillarisation markers examined (Poole et al., 2021). While speculative, potential factors include limitations in exercise‐induced arteriolar vasodilatation and/or perfusive oxygen conductance that slowed muscle oxygen delivery (Poole et al.,^2021^). In the absence of explicit criteria to verify that the single‐leg ${\dot{V}}_{O_{2}\mathrm{peak}}$ tests in fact involved a true ‘maximal’ effort at all time points (e.g., evidence of a plateau in oxygen uptake despite an increase in workload or incorporation of a verification phase), we also cannot rule out that a learning effect or some other extraneous factor contributed to the increase in single‐leg ${\dot{V}}_{O_{2}\mathrm{peak}}$ measured after the intervention.

### 4.5 Strengths and limitations

A strength of the present study was the within‐participant design and incorporation of a non‐exercise control group, which is rare in studies of this type. This model eliminates the potential for observing variable inter‐individual differences in a wide‐range of training responses in which some participants are more responsive to an exercise stimulus compared to others (Pickering & Kiely, 2019). The comparisons with a CON limb ensured that the observed changes were due to the training intervention and not confounding factors such as, but not limited to, age, sex, diet or training status. The study protocol was pre‐registered prior to data collection and included a sample size estimate that yielded a larger total sample than in previous single‐leg cycling studies on this topic (MacInnis, Zacharewicz et al., 2017; Skelly et al., 2023). As discussed above, however, the study could have been strengthened by characterising exercise intensity using a metabolic rather than opposed to percentage of *W*_peak_ given the substantial inter‐individual variability in intensity domain boundaries when expressed using the latter approach. While some of the variability in skeletal muscle responses may have been related to the way exercise intensity was prescribed, there was no relationship between baseline fitness or various indices of absolute or relative training workload and changes in specific biomarkers.

The use of the single‐leg cycling model also introduces a limitation, as it assumes that both limbs will respond similarly to the same intervention and that there is no biochemical cross‐talk between limbs (MacInnis, McGlory et al., 2017). Another limitation was the lack of a distinct exercise performance test, such as a single‐leg cycling time trial or time‐to‐fatigue test. In the present study, single‐leg *W*_peak_ during an incremental exercise test to volitional exhaustion was increased after training in the trained leg compared to pre‐training and the control leg. In contrast, single‐leg ${\dot{V}}_{O_{2}\mathrm{peak}}$ showed a main effect for time such that it was higher after training in both legs. Previously studies have also found a disconnect between training‐induced changes in single‐leg *W*_peak_ and ${\dot{V}}_{O_{2}\mathrm{peak}}$ (MacInnis, Zacharewicz et al., 2017; Skelly et al., 2023), but inclusion of a standalone performance test before and after training in both legs would have facilitated a more rigorous assessment in this regard.

### 4.6 Conclusion

In summary, the present study found that biomarkers of skeletal muscle oxidative capacity were unchanged after 6 weeks of twice‐weekly INT performed using single‐leg, counterweighted cycling in young, active adults. The lack of change in oxidative capacity may be owing to an insufficient training stimulus or to baseline participant fitness or measurement variability as indicated by the TE of ∼20%–40% for most biomarkers between legs at baseline. Future work should clarify the effect of weekly training frequency on skeletal muscle oxidative capacity in active adults while accounting for variability across biomarkers. A higher weekly training frequency may be needed to elicit responses, or a higher exercise intensity or volume is required when training frequency is limited.

## AUTHOR CONTRIBUTIONS

Emileigh R. Binet and Martin J. Gibala conceived the study. Emileigh R. Binet, Beatrice Yee, James McKendry, Changhyun Lim, Fiona J. Babir, Douglas L. Richards, and Martin J. Gibala facilitated data collection. Emileigh R. Binet performed data analyses, interpreted results, prepared figures, and wrote the initial draft of the manuscript. All authors edited, and revised the final manuscript. All authors have read and approved the final version of this manuscript and agree to be accountable for all aspects of the work in ensuring that questions related to the accuracy or integrity of any part of the work are appropriately investigated and resolved. All persons designated as authors qualify for authorship, and all those who qualify for authorship are listed.

## CONFLICT OF INTEREST

Martin J. Gibala is an advisor to and holds equity in Longevity League Ltd, a US‐based company whose services in part relate to exercise. The authors otherwise declare no conflicts of interest.

## GENERATIVE AI STATEMENT

No use of a generative artificial intelligence tool was used at any point in the generation of this manuscript.

## Data Availability

Data will be made available upon reasonable request.

## 1

**TABLE A1:** Products used for citrate synthase assays.

Product	Cat. no.	Company
Bicinchoninic acid protein assay kit (Pierce)	A55865	Thermo Fisher Scientific
Acetyl coenzyme A trisodium salt ≥93% (HPLC), powder	A2056	Sigma‐Aldrich
d‐Mannitol (≥ 98 % (GC))	M4125	Sigma‐Aldrich
EGTA (molecular biology grade)	324626	Sigma‐Aldrich
HEPES (≥ 99.5 % (titration))	H3375	Sigma‐Aldrich
Mini‐protease inhibitor tablets	4693124001	Roche
Oxaloacetic acid ≥ 97 % (HPLC)	O4126	Sigma‐Aldrich
Sucrose (for molecular biology, ≥99.5% (GC))	S0389	Sigma‐Aldrich
Tris‐hydrochloride	BP153	Thermo Fisher Scientific
5,5′‐Dithiobis (2‐nitrobenzoic acid) ≥98%	D8130	Sigma‐Aldrich

Abbreviations: GC, gas chromatography; HPLC, high‐performance liquid chromatography. *Note*Roche Diagnostics, Indianapolis, IN, USA; Sigma‐Aldrich, St Louis, MO, USA; Thermo Fisher Scientific, Waltham, MA, USA.

**TABLE A2:** Products used for western immunoblotting.

Product	Cat. No.	Company
Bicinchoninic acid protein assay kit (Pierce)	A55865	Thermo Fisher Scientific
Mini‐protease inhibitor tablets	4693124001	Roche
Molecular grade skim milk	SKI400	BioShop
PHOSStop‐phosphatase inhibitor tablets	4906845001	Roche
Ponceau S staining solution	A40000279	Thermo Fisher Scientific
Precision plus protein dual colour standards	1610374S	Bio‐Rad
Radioimmunoprecipitation assay buffer	89900	Thermo Fisher Scientific
SuperSignal West Pico PLUS chemiluminescent substrate	34577	Bio‐Rad
Trans‐Blot turbo RTA midi 0.2 µm PVDF transfer Kit	1704273	Bio‐Rad
Polysorbate 20 (Tween)	BP337	Thermo Fisher Scientific
2‐Mercaptoethanol	1670710	Bio‐Rad
4× Laemmli sample buffer	1610747	Bio‐Rad
4%–20% Criterion TGX Precast midi protein gel, 26 well, 15 µl	5671095	Bio‐Rad
10× Tris/glycine/SDS‐buffer, pH 8.3	1610732	Bio‐Rad
10× Tris‐buffered saline	1706435	Bio‐Rad

*Note*: Bio‐Rad Laboratories, Hercules, CA, USA; BioShop, Burlington, Ontario, Canada; Roche, Basel, Switzerland; Sigma‐Aldrich, St Louis, MO, USA; Thermo Fisher Scientific, Waltham, MA, USA.

Abbreviations: PVDF, polyvinylidene fluoride; RTA, ready‐to‐assemble.

**TABLE A3:** Products used for immunofluorescence.

Product	Cat. no.	Company
Bovine serum albumin	A9647	Sigma‐Aldrich
Fetal bovine serum	A3160701	Thermo Fisher Scientific
Fisherbrand feather microtome blades‐S22	12‐631‐P	Thermo Fisher Scientific
Fisherbrand premium frosted slides (25 × 75 mm)	12‐550‐15	Thermo Fisher Scientific
Fisherfinest rectangular cover glasses (24 × 50 mm, 1.5)	12‐541‐033	Thermo Fisher Scientific
Fluoromount aqueous mounting medium	F4680	Sigma‐Aldrich
Goat serum	16210‐064	Thermo Fisher Scientific
Glycerol, ≥99.0% (GC)	G7757	Sigma‐Aldrich
ImmEdge hydrophobic barrier PAP pen	H‐4000	Vector Laboratories
Paraformaldehyde, 4%	J61899.AK	Thermo Fisher Scientific
Polysorbate (Tween) 20	BP337	Thermo Fisher Scientific
Tissue‐plus optimal cutting temperature compound	23‐730‐571	Thermo Fisher Scientific
Triton X‐100, surfact‐amps detergent	85112	Thermo Fisher Scientific
10× Phosphate buffered saline	1610780	Bio‐Rad

*Note*: Bio‐Rad Laboratories, Hercules, CA, USA; Sigma‐Aldrich, St Louis, MO, USA; Thermo Fisher Scientific, Waltham, MA, USA; Vector Laboratories, Newark, CA, USA.

Abbreviations: GC, gas chromatography; HPLC, high‐performance liquid chromatography.
